# *Salvia officinalis* restores semen quality and testicular functionality in cadmium-intoxicated male rats

**DOI:** 10.1038/s41598-023-45193-1

**Published:** 2023-11-27

**Authors:** Wesam T. Basal, Aliaa M. Issa, Omnia Abdelalem, Amel R. Omar

**Affiliations:** Department of Zoology, Faculty of Science, Cairo University, Giza, 12613 Egypt

**Keywords:** Cell biology, Developmental biology, Molecular biology, Zoology

## Abstract

The present study investigated the potential ability of *Salvia officinalis*, one of the oldest medicinal plants, to protect male rats against cadmium reproductive toxicity. Twenty-eight healthy male rats were randomly allocated into four groups (n = 7); control, *Salvia*-extract treated group, cadmium treated group and a group treated with both Cd and *Salvia*. Administration of cadmium reduced the relative testis to body weight and significantly affected sperm parameters by decreasing motility, viability, count and increasing morphological aberrations. Comet assay was used to detect DNA fragmentation in sperms of the rats exposed to Cd. Serum levels of testosterone T, follicle stimulating hormone FSH, and luteinizing hormone LH were significantly decreased. The biochemical analysis of testicular tissue showed a significant rise in Malondialdehyde MDA level coupled with a decrease in the activity of antioxidant enzymes (superoxide dismutase SOD, glutathione peroxidase GPx and catalase CAT). The histological examination of testis sections after Cd administration revealed severe degeneration of spermatogenic cells. Seminiferous tubules were filled with homogenous eosinophilic fluid associated with atrophy of other seminiferous tubules. Co-treatment with the *Salvia officinalis* extract restored the oxidative enzymes activities and decreased the formation of lipid peroxidation byproduct, which in turn ameliorated the effect of Cd on sperm parameters, DNA damage and testis histopathology. Taken together, it can be concluded that the synergistic antioxidant and radical savaging activities of *Salvia officinalis* prevented the effect of Cd on semen quality, sperm DNA damage, along with the oxidative stress and histological abnormalities in the testis tissues.

## Introduction

Despite revealing many potential root causes for male infertility, the etiology of about 50% of the cases remained obscure^1^. Exposure to environmental pollutants, including heavy metals, has been linked to male infertility^2^. Several studies on animal models (specially rodents) and the data collected from human epidemiological studies proved that cadmium (Cd) exposure induces various reproductive health impairments^3^. It affects male fertility through several routes: by decreasing sperm motility and viability, undermining the endocrine function, impairing spermatogenesis, inducing structural damage of testicular tissue, and sabotage function in Sertoli and Leydig cells^4–7^.

Some recent studies showed that the bioactive components of *Salvia officinalis,* one of the oldest used medicinal plants, have potent anti-inflammatory and antioxidant effects^8,9^ which might result in alleviating the reproductive toxicity of Cd that works mainly through inflammatory and oxidative stress mechanisms^4^.

Traditional semen analysis is based on sperm concentration, motility, and morphology^10,11^. Semen examination of poorly fertile males usually reveals sperms with low count, decreased motility, or abnormal morphology^12^. Strikingly, 15% of males with normal traditional analysis profiles still showed fertility problems. Therefore, to duly diagnose male infertility, it may be necessary to measure other sperm parameters^13^.

DNA fragmentation is an important factor in the etiology of male infertility. However, its inclusion in routine semen analysis is still under evaluated. DNA fragmentation was shown to be a major indicator of fertility potential, even more than conventional semen parameters^13^. Comet assay for sperms or testicular cells have been applied in several studies to evaluate effect of reproductive toxins and genotoxins in male human and rats^14,15^.

Serum levels of FSH, LH and T are used as good indicators of fertility status^16,17^. Low serum levels of these hormones were previously discovered in male rats exposed to cadmium^18^.

Histopathological evaluation provides one of the most sensitive methods to identify the probable target cell. Careful examination of the earliest morphological and microscopical changes shed some light on the possible mechanisms of male reproductive system toxicants^19,20^.

The morphological and molecular damage imposed by Cd in testicular tissue were mainly attributed to the oxidative stress^4,21^. For assessment of the oxidative stress in testicular tissue, the levels of malondialdehyde (MDA) and the enzyme activities of catalase (CAT), glutathione peroxidase (GPx) and superoxide dismutase (SOD) are analyzed spectrophotometrically^22,23^. The current study aims at determining the potential ameliorative role of *Salvia officinalis* against Cd-induced reproductive toxicity in rats.

## Materials and methods

### Materials

Cadmium chloride (CdCl_2_) was purchased from Sigma-Aldrich (St. Louis, MO, USA). High quality *Salvia officinalis*, commonly known as Sage, was purchased from a local shop.

### Preparation and identification of *Salvia officinalis* extract

The bioactive components of the leaves of *Salvia officinalis* were extracted with methanol at room temperatures using the protocol mentioned by Alkan et al.^24^. The extract was analysed for phenol content using High Performance Liquid Chromatography (HPLC) (Agilent 1260 infinity, Waldbronn, Germany). All experiments and procedures comply with the IUCN Policy Statement on Research Involving Species at Risk of Extinction and the Convention on the Trade in Endangered Species of Wild Fauna and Flora.

### Experimental animals

Twenty-eight adult male Wistar rats of 8 weeks old (weighing 180–200 g) were purchased from the Experimental Animal Research Center, Faculty of Pharmacy, Kafr El sheikh University, Egypt. The animals were maintained in a climate-controlled room under a 12-h alternating light/dark cycle, 20.1 to 21.2 °C temperature and 50 to 55.5% relative humidity. The rats were fed standard chow and water ad libitum. All experiments and procedures were performed in accordance with ARRIVE guidelines and were consented by The Institutional Animal Care and Use Committee (IACUC), Faculty of Science, Cairo University with the approval number (CU/I//F/14/23). All experiments were performed in accordance with relevant guidelines and regulations.

### Experimental design

Wistar rats were allotted into four groups, each with seven rats as follows:

Group 1 was injected intraperitoneally (i.p.) with saline (vehicle of CdCl_2_) and gauged with water (vehicle of *Salvia officinalis*) to serve as the control group. Group 2 orally received 200 mg/kg of the methanolic extract of *Salvia officinalis* daily for 8 weeks via an oral tube^25^. Group 3 received a single dose of 1 mg/kg of CdCl_2_ dissolved in saline solution through i.p. injection, according to Chen et al.^26^. Group 4 received a single i.p. dose of Cd as in group 3 simultaneously with the 8 weeks extract treatment as in group 2.

### Semen analysis

#### Sperm motility and count

Epididymal suspension with sperms was poured into counting chamber of the hemocytometer, covered with cover slip, and examined under a phase-contrast microscope with a warming plate at 40× magnification. Sperm motility was calculated according to the procedure described by El-Magd et al.^27^ and sperm count according to Yokoi et al.^28^. Approximately, five fields per sample were randomly examined.

#### Sperm viability and morphology

Sperm viability and abnormalities were examined using eosin-nigrosin (EN) staining (Sigma-Aldrich, Saint Louis, USA). A minimum of 200 spermatozoa were analyzed from each animal under 400× magnification with bright-field microscopy and the proportion of viable sperm cells and morphologically abnormal spermatozoa was recorded according to the criteria described by Okamura et al.^29^.

### Assessment of DNA fragmentation by comet assay (single cell gel electrophoresis, SCGE)

A sperm cell pellet (about 1 g) was homogenized in 1 ml of cold mincing solution then suspended in darkness. The extent of DNA strand breaks in control and treated cells of both cell lines was assessed using the alkaline comet assay, as previously described by Tice et al*.* (2000)^30^. Comets were analyzed using Komet 5.0 analysis system developed by Kinetic Imaging, Ltd. (Liverpool, United Kingdom) connected to a charge-coupled device (CCD) camera and 40× objective of fluorescent microscope with excitation filter 420–490 nm (issue 510 nm).

### Hormonal assay

Levels of serum hormones were measured using commercial ELISA kits for luteinizing hormone (LH) (Cat. No. BC-1029, BioCheck, Foster city, CA, USA), follicle-stimulating hormone (FSH) (Cat. No. RH-251, DSI, Milan, Italy), and testosterone (Cat. No. CAN-TE- 250, DBC, Ontario, Canada). The procedures were carried out following the manufacturer's protocol and the absorbance was measured at 450 nm.

### Estimation of oxidative stress markers

The effect of exposure to cadmium and/or *Salvia officinalis* on oxidative stress markers in the testicular tissue were measured colorimetrically using commercial kits (Bio-diagnostic, Cairo, Egypt). The procedures were carried out according to the manual provided by the manufacturer to measure the level of malondialdehyde (MDA) (Cat. No. MD 25 29) and the activities of superoxide dismutase (SOD) (Cat. No. SD 25 21), Catalase (CAT) (Cat. No. CA 25 17), and glutathione peroxidase (GPx) (Cat. No. GP 2524).

### Histopathological studies

Testis tissue samples from the testis were immediately removed from the euthanized rats and fixed using 10% neutral buffered formalin (Sigma-Aldrich, Saint Louis, USA), sectioned, and stained with Hematoxylin and Eosin (Sigma-Aldrich, Saint Louis, USA) for routine examination^31^. Histopathological quantitative scoring was assessed according to Venditti et al.^32^. Testicular tissue was evaluated using the image analysis system Leica QWin DW3000 (LEICA Imaging Systems Ltd., Cambridge, England) for tubular diameter, epithelium thickness, lumen diameter, spermatic cells degeneration, atrophied seminiferous tubules, and seminiferous tubules with oedema. A comprehensive count of 175 seminiferous tubules per group (25/animal) was conducted.

### Statistical analysis

Data were analyzed using statistical software package (IBM-SPSS) version 23 software. Kolmogorov–Smirnov test showed that the raw data were normally distributed. One-way ANOVA was applied to study the effect of treatment on the studied parameters. Least significant difference (LSD) test was used to illustrate the statistical differences among the experimental groups. Data is displayed as mean ± standard error of mean (Supplementary table).

## Results

### Identification of* Salvia officinalis* methanolic extract using HPLC

**Figure 1:**
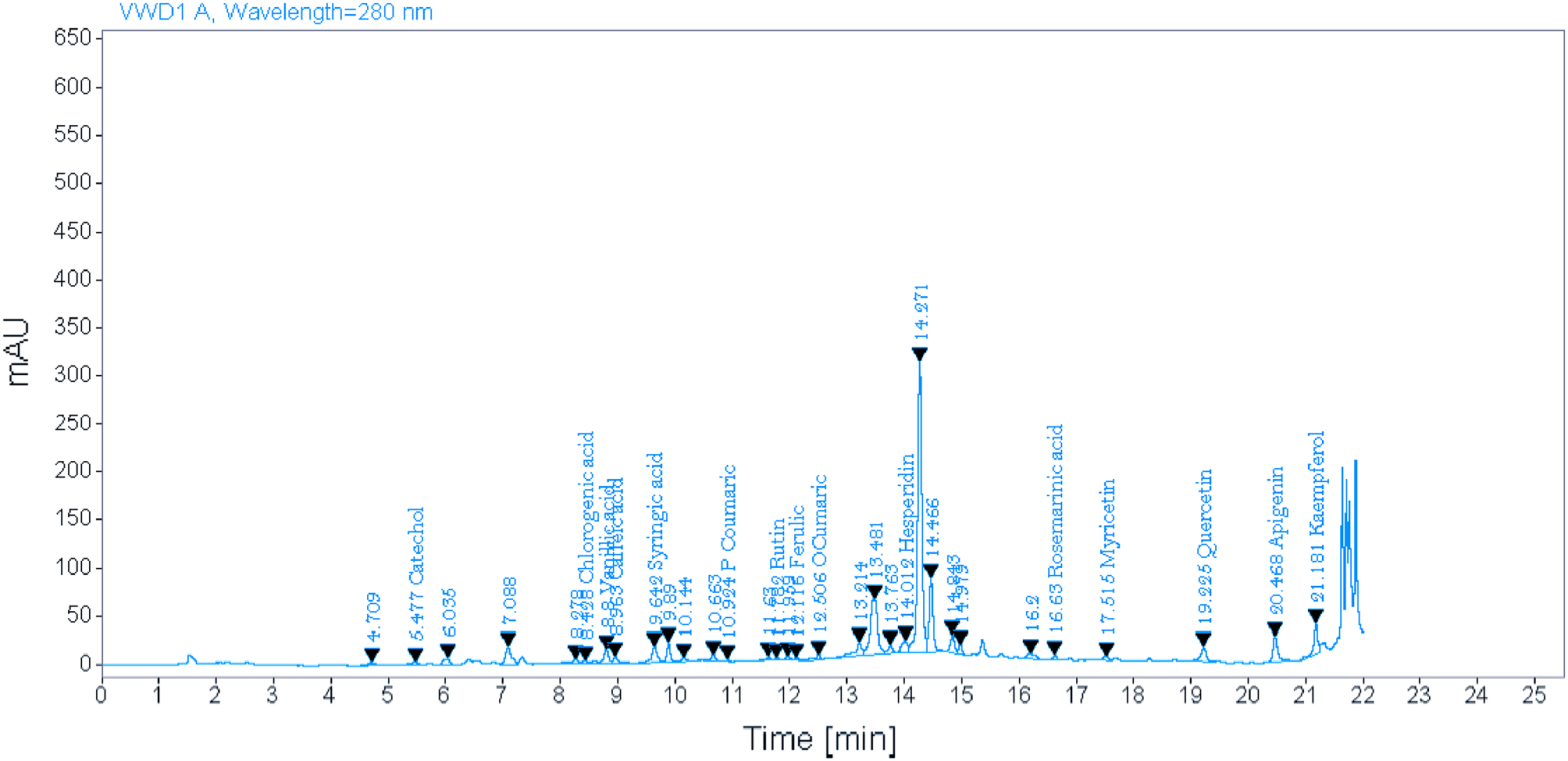
HPLC analysis for methanolic extract of *Salvia officinalis.*

HPLC for the methanolic extract of *Salvia officinalis* verified the presence of 15 phenolic compounds: catechol, chlorogenic acid, vanillic acid, caffeic acid, syringic acid, p-coumaric acid, rutin, ferulic, o-coumaric acid, hesperidin, rosemarinic acid, myricetin, quercetin, apigenin, and kaempferol (Fig. 1).

### Relative testis to body weight (g/g)

The examined rats of all the four groups (control group, *Salvia officinalis* treated group, Cd treated group, Cd + *Salvia* treated group) did not show any obvious sign of toxicity or abnormal behavior. However, rats of group 3 and 4 recorded a slightly less body weight gain and a notable decrease in testis weight. As shown in Table. 1, the relative testis weight in Cd group (0.005 ± 0.0005) was significantly decreased (*P* ≤ 0.05) compared to the control group (0.015 ± 0.001). Rats treated with *Salvia* extract showed no significant change in relative testis weight (0.017 ± 0.001) compared to the control group. The co-administration of *Salvia* and Cd showed a significant testis weight ratio decrease (0.010 ± 0.001) compared to the control group, and a significant increase when compared to the average weight recorded in Cd group.

**Table 1:** Average testis to body weight for all rats in the four studied groups.

Group	Mean of relative testis/body weight (g/g)
Control (G1)	0.015 ± 0.001
*Salvia officinalis* extract treated group (G2)	0.017 ± 0.001
	P1 = 0.18
Cadmium treated group (G3)	0.005 ± 0.0005*
	P1 = 0.00
Cadmium and extract treated group (G4)	0.010 ± 0.001*^#^
	P1 = 0.02, P2 = 0.01

*Significant difference (P1), as compared to the control group.

^#^Significant difference (P2), as compared to the Cd-treated group.

### Semen analysis

#### Sperm motility (%)

The percentage of sperm motility in the control group was 91.25 ± 3.27. In the rats treated with *Salvia* extract, the sperm motility was 90.42 ± 3.54 showing no significant change compared to the control group. In the group treated with cadmium, the recorded sperm motility (62.31 ± 2.26) was significantly decreased (*P* ≤ 0.05) compared to the control one. The co-administration of *Salvia* with Cd showed a significant decrease in sperm motility (76.27 ± 3.12) compared to the control group, and a significant increase when compared to the percentage of sperm motility recorded in Cd group (Table 2).

**Table 2:** Effect of cadmium or/and *Salvia officinalis* on sperm parameters in the semen collected from all rats in the four studied groups.

Group	Mean of percentage of sperm motility (%)	Mean of sperm count (10^6^/ml)	Mean of percentage viability of sperm (%)	Mean of sperm abnormalities (%)
Control (untreated) (G1)	91.25 ± 3.27	74.48 ± 5.02	93.37 ± 3.1	8.66 ± 0.52
*Salvia officinalis* extract treated group (G2)	90.42 ± 3.54	72.56 ± 4.11	92.42 ± 3.54	8.15 ± 0.46
	P1 = 0.86	P1 = 0.73	P1 = 0.83	P1 = 0.68
Cadmium treated group (G3)	62.31 ± 2.26*	36.51 ± 2.32*	62.31 ± 2.26*	20.28 ± 1.40*
	P1 = 0.00	P1 = 0.00	P1 = 0.00	P1 = 0.00
Cadmium and extract treated group (G4)	76.27 ± 3.12*^#^	51.6 ± 3.05*^#^	76.27 ± 3.12*^#^	14.36 ± 0.72*^#^
	P1 = 0.01, P2 = 0.01	P1 = 0.00, P2 = 0.02	P1 = 0.00, P2 = 0.01	P1 = 0.00, P2 = 0.00

*Significant difference (P1), as compared to the control group.

^#^Significant difference (P2), as compared to the Cd-treated group.

#### Sperm count (10^6^/ml)

The mean value of sperm count in semen samples of rats in the control group was 74.48 ± 5.02. In the group treated with *Salvia* extract, the mean value of sperm count was 72.56 ± 4.11 showing no significant change compared to the control group. In the group treated with cadmium, the recorded sperm count mean (36.51 ± 2.32) was significantly decreased (*P* ≤ 0.05) compared to the control group. Despite showing a significant decrease in sperm count (51.6 ± 3.05) in the fourth group (treated with Cd and *Salvia*) compared to the control group, the value was significantly higher than the mean value of sperm count recorded in Cd treated group (Table 2).

#### Sperm viability (%)

The percentage of sperm viability in the control group was 93.37 ± 3.1. In the group treated with *Salvia* extract, the viability percentage was 92.42 ± 3.54 showing no significant change compared to the control group. In the group treated with cadmium, the recorded viability percentage (62.31 ± 2.26) was significantly decreased (*P* ≤ 0.05) compared to the control group. The co-administration of *Salvia* with Cd showed a significant decrease in percentage of viability (76.27 ± 3.12) compared to the control group, and a significant increase when compared to the sperm viability recorded in Cd exposed group (Table 2).

#### Sperm abnormalities (%)

The recorded percentage of sperm abnormalities in control group was 8.66 ± 0.52. In the group treated with *Salvia* extract, the percentage of sperm abnormalities was 8.15 ± 0.46 showing no significant change compared to the control group. In the group treated with cadmium, the recorded percentage of sperm abnormalities (20.28 ± 1.4) was significantly increased (*P* ≤ 0.05) compared to the control group. In *Salvia* + Cd group showed a significant decrease in percentage of sperm abnormalities (14.36 ± 0.72) compared to Cd group. However, it was still significantly higher than the percentage of sperm abnormalities recorded in the control group (Table 2).

### Assessment of DNA fragmentation in sperms by comet assay (Single cell gel electrophoresis (SCGE)

**Figure 2:**
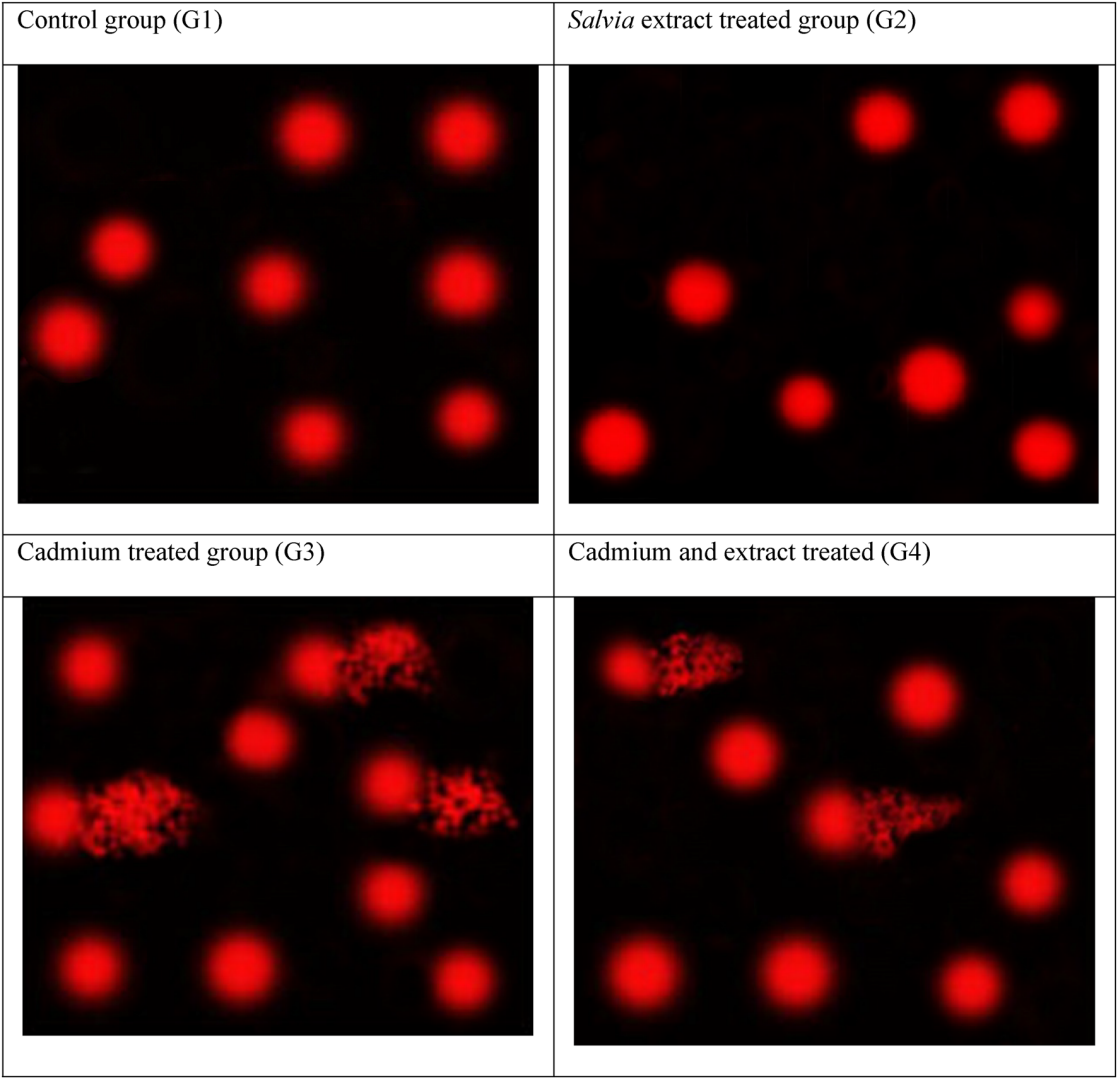
Photomicrographs showing the extent of DNA damage in sperms of rats in all groups as detected by comet assay. G1: Control, G2: *Salvia* extract treated group, G3: Cadmium treated group, G4: Cadmium and extract treated group.

In *Salvia* extract treated group, the average tail length was 2.11 ± 0.10 showing no significant change compared to the control group (1.62 ± 0.12). In the group treated with cadmium, the recorded average tail length (8.35 ± 0.31) was significantly increased (*P* ≤ 0.05) compared to the control group. The co-administration of *Salvia* + Cd showed a significant increase in tail length (7.03 ± 0.23) when compared to the control group, and a significant decrease when compared to Cd group (Table 3, Fig. 2).

**Table 3:** Comet assay parameters obtained by image analysis for sperms collected from all rats in the four groups.

Group	Tailed %	Untailed %	Tails length µm	Tail DNA%	Tail moment (arbitrary units)
Control (untreated) (G1)	2	98	1.62 ± 0.12	1.64 ± 0.05	2.66 ± 0.03
*Salvia officinalis* extract treated group (G2)	3	97	2.11 ± 0.10	1.99 ± 0.11	4.20 ± 0.12
			P1 = 0.13	P1 = 0.14	P1 = 0.46
Cadmium treated group (G3)	25	75	8.35 ± 0.31*	6.93 ± 0.22*	57.86 ± 2.23*
			P1 = 0.00	P1 = 0.00	P1 = 0.00
Cadmium and extract treated group (G4)	18	82	7.03 ± 0.23*^#^	5.28 ± 0.16*^#^	37.12 ± 1.67*^#^
			P1 = 0.00, P2 = 0.00	P1 = 0.00, P2 = 0.00	P1 = 0.00, P2 = 0.00

*Significant difference (P1), as compared to the control group.

^#^Significant difference (P2), as compared to the Cd-treated group.

### Serum hormonal levels

The average serum concentrations of FSH, LH and testosterone in the control group (G1) were 6.21 ± 0.31 U/ml, 33.48 ± 0.56 U/ml, and 7.52 ± 0.59 ng/ml respectively. The administration of *Salvia* extract alone did not significantly affect the serum levels of any of the hormones recording 6.83 ± 0.27 U/ml for FSH, 32.17 ± 0.58 U/ml for LH, and 6.93 ± 0.64 ng/ml for T. The cadmium administration resulted in a significant decrease in the serum concentration levels of all of the three hormones compared to the control group recording 3.36 ± 0.16 U/ml for FSH, 24.33 ± 0.36 U/ml for LH, and 1.45 ± 0.08 ng/ml for T. Meanwhile, in *Salvia* + Cd group, despite recording serum level concentrations significantly higher than those recorded for Cd group (5.02 ± 0.19 U/ml, 28.42 ± 0.31 U/ml, and 4.04 ± 0.24 ng/ml) respectively, the values were still significantly less than those recorded in the control group (Table 4).

**Table 4:** Levels of follicle stimulating hormone (FSH), Luteinizing hormone (LH) and testosterone (T) in serum samples collected from all rats in the four studied groups.

Group	Means of serum level of FSH (U/ml)	Means of serum level of LH (U/ml)	Means of serum level of T (ng/ml)
Control (G1)	6.21 ± 0.31	33.48 ± 0.56	7.52 ± 0.59
*Salvia officinalis* extract treated group (G2)	6.83 ± 0.27	32.17 ± 0.58	6.93 ± 0.64
	P1 = 0.11	P1 = 0.08	P1 = 0.38
Cadmium treated group (G3)	3.36 ± 0.16*	24.33 ± 0.36*	1.45 ± 0.08*
	P1 = 0.00	P1 = 0.00	P1 = 0.00
Cadmium and extract treated group (G4)	5.02 ± 0.19*^#^	28.42 ± 0.31*^#^	4.04 ± 0.24*^#^
	P1 = 0.01, P2 = 0.00	P1 = 0.00, P2 = 0.00	P1 = 0.00, P2 = 0.00

*Significant difference (P1), as compared to the control group.

^#^Significant difference (P2), as compared to the Cd-treated group.

### Detection of oxidative stress markers

The average level of MDA in the testicular tissue of control group rats was 15.11 ± 0.74 nmol/g tissue. While in the group treated with *Salvia* extract, the average level of MDA was 14.02 ± 0.77 nmol/g tissue showing no significant change compared to the control group. In the group treated with cadmium, the recorded average MDA level (28.48 ± 1.25 nmol/g tissue) was significantly increased (*P* ≤ 0.05) compared to the control group. The co-administration of *Salvia* showed a significant increase in MDA level (19.35 ± 0.92 nmol/g tissue) compared to the control group, and a significant decrease when compared to the average MDA-level recorded in Cd group (Table 5).

**Table 5:** Malondialdehyde (MDA) levels in testes of rats in the four studied groups (nmol/g tissue).

Group	Means of testicular levels of MDA (nmol/g)
Control (G1)	15.11 ± 0.74
*Salvia officinalis* extract treated group (G2)	14.02 ± 0.77
	P1 = 0.44
Cadmium treated group (G3)	28.48 ± 1.25*
	P1 = 0.00
Cadmium and extract treated group (G4)	19.35 ± 0.92*^#^
	P1 = 0.01, P2 = 0.00

*Significant difference (P1), as compared to the control group.

^#^Significant difference (P2), as compared to the Cd-treated group.

The average testicular levels of superoxide dismutase (SOD), catalase (CAT) and glutathione peroxidase (GPx) in the control group rats were (1117.26 ± 74.5, 251.6 ± 74.5 and 12.41 ± 0.72 U/g tissue) respectively. In the group exposed to *Salvia* extract alone, the testicular levels of the enzymes were not significantly affected recording 1206.93 ± 71.34 U/g for SOD, 278.75 ± 15.36 U/g for CAT, and 13 ± 0.75 U/g for GPx. The testis tissue of the cadmium group showed a significant decrease in the activities of the three enzymes compared to the control group recording 648.6 ± 40.37 U/g for SOD, 128.17 ± 6.42 U/g for CAT, and 7.14 ± 0.37 U/g for GPx. The co-administration of *Salvia* + Cd despite recording testicular activities significantly less than the control group (906.34 ± 52.65, 182.54 ± 8.7, and 10.25 ± 0.42 U/g tissue) respectively, they were significantly higher than the Cd group testicular activities (Table 6).

**Table 6:** The mean value for testicular activity of superoxide dismutase (SOD), catalase (CAT) and glutathione peroxidase (GPx) in the four studied groups (U/g tissue).

Group	SOD (U/g tissue)	CAT (U/g tissue)	GPx (U/g tissue)
Control (G1)	1117.26 ± 74.5	251.6 ± 13.49	12.41 ± 0.72
*Salvia officinalis* extract treated group (G2)	1206.93 ± 71.34	278.75 ± 15.36	13.00 ± 0.75
	P1 = 0.33	P1 = 0.14	P1 = 0.50
Cadmium treated group (G3)	648.6 ± 40.37*	128.17 ± 6.42*	7.14 ± 0.37*
	P1 = 0.00	P1 = 0.00	P1 = 0.00
Cadmium and extract treated group (G4)	906.34 ± 52.65*^#^	182.54 ± 8.70*^#^	10.25 ± 0.42*^#^
	P1 = 0.04, P2 = 0.02	P1 = 0.00, P2 = 0.01	P1 = 0.03, P2 = 0.01

*Significant difference (P1), as compared to the control group.

^#^Significant difference (P2), as compared to the Cd-treated group.

### Histopathology of testis tissue

**Figure 3:**
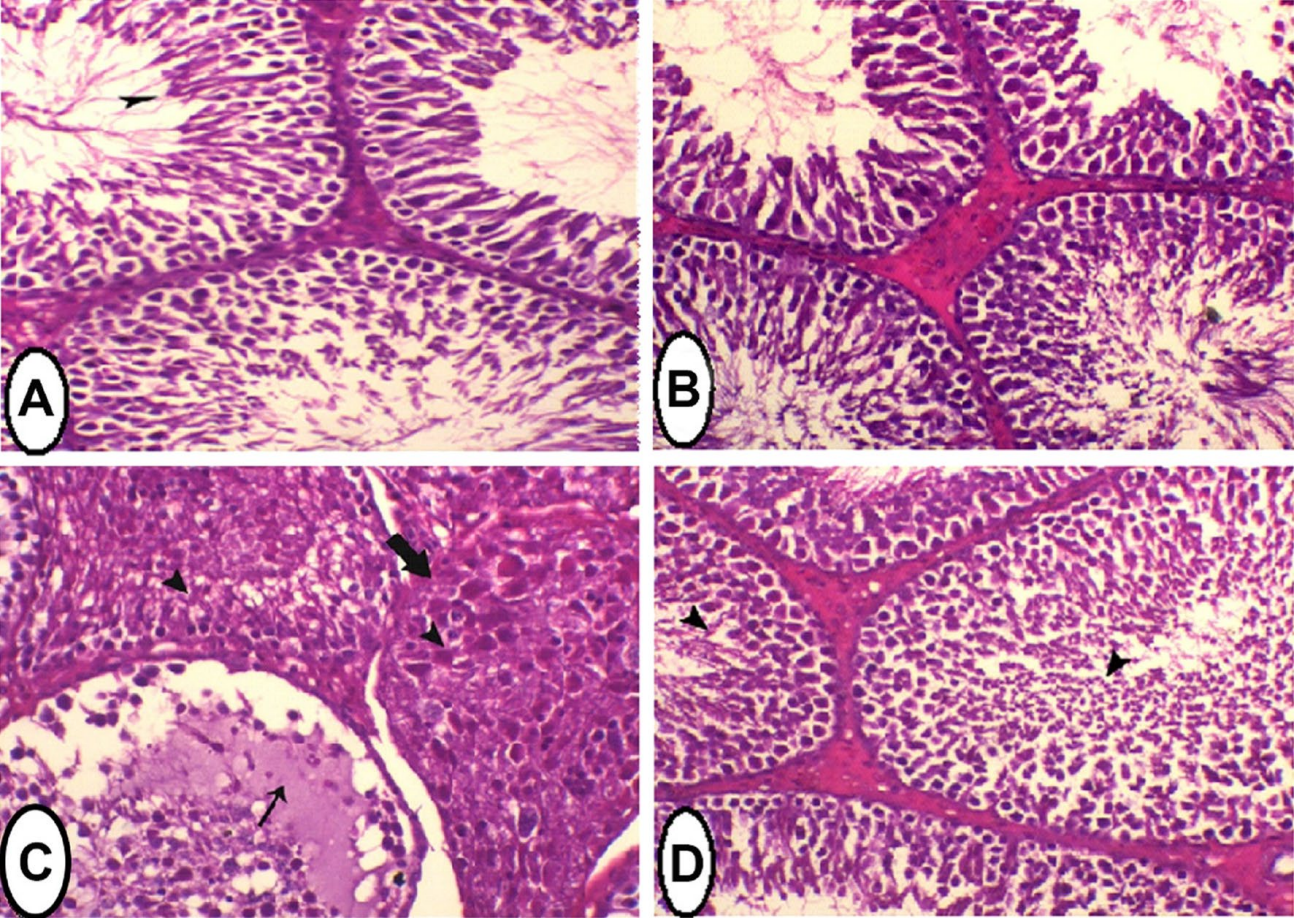
Sections in testis of rats of all the four groups stained with H & E. X = 400. (**A**) Testis of control (normal) rat showing normal seminiferous tubules with active spermatogenesis, arrowhead indicates free sperm. (**B**) Testis of rat treated with *Salvia* extract showing several seminiferous tubules within the normal limits. (**C**) Testis of rat treated with cadmium showing severe degeneration of spermatogenic cells (arrowhead). Seminiferous tubules were filled with homogenous eosinophilic fluid (thin arrow) associated with atrophy of other seminiferous tubules (thick arrow). (**D**) Testis of cadmium-intoxicated rat treated with *Salvia* extract showing improvement in spermatogenesis accompanied with seminiferous tubules regeneration with only mild spermatid layer degeneration (arrowheads).

In the control group, the testis sections showed normal appearance of seminiferous tubules, spermatogenic cells with active spermatogenesis and normal interstitial cells. Sections taken from rats treated with *Salvia* extract showed an increase in the number of seminiferous tubules within the normal limits. In Cd treated group, the testis sections showed severe degeneration of spermatogenic cells. Seminiferous tubules were filled with homogenous eosinophilic fluid associated with complete atrophy of some of the seminiferous tubules. These histopathological changes were reduced after co-administration of *Salvia*, and the testes tissue showed improvements in spermatogenesis accompanied with seminiferous tubules regeneration with only mild spermatid layer degeneration (Fig. 3). The histological examination was validated by the recording of six morphometric parameters (Table 7). The tubular diameter, epithelial thickness, lumen diameter, numbers of degenerated spermatogenic cells, atrophied seminiferous tubules, and seminiferous tubules with eosinophilic fluid in the control group (G1) were 278.49 ± 0.72, 187.41 ± 2.65, 91.08 ± 2.74, 5.43 ± 0.48, 00.00 ± 0.00, and 00.00 ± 0.00, respectively. The administration of *Salvia* extract alone (G2) did not significantly affect these parameters recording 274.02 ± 1.14 for tubular diameter, 180.67 ± 3.16 for epithelial thickness, 93.35 ± 3.77 for lumen diameter, 8.86 ± 0.63 for the number of degenerated spermatogenic cells, and 00.00 ± 0.00 for the numbers of atrophied seminiferous tubules and seminiferous tubules with eosinophilic fluid. In Cd treated group, a significant decrease in the tubular diameter (221.99 ± 9.87) and epithelial thickness (98.24 ± 2.06) was recorded along with a non-significant increase in lumen diameter (123.76 ± 10.89). Moreover, the numbers of degenerated spermatogenic cells (109.29 ± 3.62), atrophied seminiferous tubules (4.71 ± 0.57), and seminiferous tubules with eosinophilic fluid (3.29 ± 0.36) were significantly elevated compared to the control group. On the other hand, the Cd + *Salvia* treated group demonstrated a significant increase in tubular diameter and epithelial thickness (244.67 ± 3.52 and 146.23 ± 5.62, respectively) compared to Cd group but still significantly lower than control group. Despite the significant reduction in the lumen diameter (98.44 ± 7.64), numbers of degenerated spermatogenic cells (32.29 ± 3.15), atrophied seminiferous tubules (1.29 ± 0.18), and seminiferous tubules with eosinophilic fluid (1.14 ± 0.26) compared to cadmium group, these values were still significantly higher than those recorded in control group.

**Table 7:** Testicular morphometric parameters from all rats in the four studied groups.

Group	Tubular diameter (µm)	Epithelium thickness (µm)	Lumen diameter (µm)	Number of degenerated spermatogenic cells	Number of atrophied seminiferous tubules	Number of seminiferous tubules with eosinophilic fluid
Control (untreated) (G1)	278.49 ± 0.72	187.41 ± 2.65	91.08 ± 2.74	5.43 ± 0.48	00.00 ± 0.00	00.00 ± 0.00
*Salvia officinalis* extract treated group (G2)	274.02 ± 1.14	180.67 ± 3.16	93.35 ± 3.77	8.86 ± 0.63	00.00 ± 0.00	00.00 ± 0.00
	P1 = 0.93	P1 = 0.57	P1 = 0.99	P1 = 0.75	P1 = 1.00	P1 = 1.00
Cadmium treated group (G3)	221.99 ± 9.87*	98.24 ± 2.06*	123.76 ± 10.89*	109.29 ± 3.62*	4.71 ± 0.57*	3.29 ± 0.36*
	P1 = 0.00	P1 = 0.00	P1 = 0.002	P1 = 0.00	P1 = 0.00	P1 = 0.00
Cadmium and extract treated group (G4)	244.67 ± 3.52*^#^	146.23 ± 5.62*^#^	98.44 ± 7.64^#^	32.29 ± 3.15*^#^	1.29 ± 0.18*^#^	1.14 ± 0.26*^#^
	P1 = 0.001, P2 = 0.03	P1 = 0.00, P2 = 0.00	P1 = 0.8, P2 = 0.08	P1 = 0.00, P2 = 0.00	P1 = 0.03, P2 = 0.00	P1 = 0.007, P2 = 0.00

*Significant difference (P1), as compared to the control group.

^#^Significant difference (P2), as compared to the Cd-treated group.

## Discussion

In the current study, administration of Cd reduced relative testis to body weight in the treated male rats. The decreased testis weight might be attributed to the necrotic and degenerative cadmium-induced changes^7^. Similar results were found by Blanco et al.^20^, when they found a significant decrease in testis weight of mice after cadmium exposure. We also found that the co-administration of *Salvia officinalis* ameliorated the effect of Cd, and the testis weight was significantly increased. Similar results were obtained by Chouabia et al.^33^ against cypermethrin- induced reprotoxicity in male Wistar rats. This weight gain might be due to the regulation of signal transduction pathways of cell growth and proliferation by the phenolic compounds of *Salvia officinalis*^34^.

We found that the administration of Cd resulted in a significant decrease in sperm count, motility, viability and altered sperm morphology. These results are consistent with the fact that heavy metals cause degenerative changes in the seminiferous tubules with loss of spermatogenesis^35,36^. Several previous studies have reported decrease in sperm count, motility and viability of mice, rats, and humans as a result of exposure to cadmium^37–40^. However, *Salvia officinalis* extract significantly improved sperm parameters when administrated with Cd. In line with our findings, Al-chalabi et al.^41^ discovered that *Salvia officinalis* causes a significant elevation in sperm activity along with a significant reduction in sperm mortality and abnormalities in diabetic male albino rats. This increase might be attributed to ability of *Salvia officinalis* extract to stimulate the growth of testes and enhance the proliferation, maturation, and differentiation of spermatozoa due to the presence of saponin and alkaloids^42,43^.

In the current study, exposure to cadmium caused significant DNA damage as detected by the single-cell gel electrophoresis assay. In a previous study, Comet assay in epididymal sperms of adult male SD rats demonstrated a significant difference in the lengths of the head and comet in all the three Cd treated groups, indicating damage in the heritable DNA^44^. In our study we observed that administration of *Salvia officinalis* with Cd protects the sperm cells from DNA damage. The protective effect of *Salvia officinalis* is consistent with our previous findings of the ability of *Salvia officinalis* to increase the levels of oxidative stress enzymes and reduce the level of MDA. Our results are in accordance with the findings of Aherne et al.^45^, who described the DNA-protective activity of *Salvia officinalis* against H_2_O_2_-induced DNA damage in Caco-2 cells. Kozics et al.^46^ showed that *Salvia officinalis* protects against oxidative stress and DNA damage through the elevation of glutathione peroxidase activity.

Our study revealed that cadmium administration resulted in a significant decrease in the circulating levels of follicle stimulating hormone (FSH), luteinizing hormone (LH) and testosterone (T). Similar results were found in previous work as they reported that cadmium significantly reduced the levels of LH, FSH and T in blood of rats^47,48^. They explained their finding that cadmium is an endocrine disruptor that interferes with the synthesis and/or regulation of the circulating levels of several hormones, including T, FSH, and LH^4,48^. The co-administration of *Salvia officinalis* with Cd increased the circulating levels of LH, FSH and testosterone. In consistency with our results, previous studies found that the level of T, LH and FSH were significantly increased in the serum of albino rats after treatment with *Salvia officinalis*^41,49^.

Upon administration of Cd, we recorded a significant increase in MDA level and a significant decrease in activities of antioxidant enzymes (SOD, GPx and CAT) which might be due to the inactivation of cellular antioxidants by the lipid peroxides and reactive oxygen species (ROS) produced by Cd intoxication^39,50^. It was also reported that Cd might disrupt the expression of these enzymes during the transcriptional stage^51^. Same results were recorded in the liver of female albino rats after Cd administration^52^. We also found that co-administration of *Salvia officinalis* with Cd led to a decrease in MDA level and increased the activities of SOD, GPx and CAT. These results agree with previous studies where they concluded that flavonoids and vanillin components of *Salvia officinalis* mediated the lipid peroxidation reduction because of their antioxidant properties and their ability of removing free radicals and chelating divalent cations^53–55^.

Upon examining the testis’s tissues, we found that administration of cadmium caused several histopathologic changes. The examination revealed severe degeneration of spermatogenic cells. Seminiferous tubules were filled with homogenous eosinophilic fluid associated with atrophy of some seminiferous tubules. Similar findings were described in previous studies where they found that Cd treatment caused degenerative changes, severe necrosis of seminiferous tubules and absence of spermatogenic cells^39,56^. Various studies have reported that testicular tissue is more vulnerable to Cd toxicity than any other organ^57^. The described histopathological damage might be attributed to the generation of ROS and oxidative stress induced by cadmium^58^ which in turn damages DNA and causes lipid and protein peroxidation^59^. When we co-administrated *Salvia officinalis* with Cd, the tissue examination showed a normal overall structure of the testes, regeneration of seminiferous tubules, and improvement in spermatogenesis compared to Cd group. Studies of Chouabia et al.^33^ and Al-Syaad^60^ showed similar results, as they found improvement in testicular histopathology of rats exposed to cypermethrin or doxorubicin after administration of *Salvia officinalis*. Bioactive constituents in *Salvia officinalis* extract have antioxidant and radical scavenging activities, which account for the plant's protective effect^52^.

## Conclusion

This study concludes that cadmium exposure negatively affects the integrity and quality of the sperm and the levels of reproductive hormones as well as testicular Oxidative stress markers and histology. *Salvia officinalis* opposes the toxic effects of cadmium exposure by significant improvement of spermatogenesis, oxidative stress, DNA fragmentation, and testicular histopathology which contributes to increasing the reproductive activity.

### Supplementary Information

Supplementary Tables.

## Data Availability

Data supporting this study are included within the article.
