# Supplementary material for: Salvia officinalis restores semen quality and testicular functionality in cadmium-intoxicated male rats
Source: Sci Rep. 2023 Nov 27;13:20808. doi: 10.1038/s41598-023-45193-1 (PMC10682483; doi:10.1038/s41598-023-45193-1)
Supplement: Supplementary file 1 — Supplementary Tables. [file 41598_2023_45193_MOESM1_ESM.docx]

Table 1. One-way ANOVA to study the effect of treatment type on the studied semen quality and hormonal levels.

|  |  | Sum of Squares | df | Mean Square | Fcalculated | P-value |
| --- | --- | --- | --- | --- | --- | --- |
| Testis (g) | Treatment | 0.000 | 3 | 0.000 | 22.087 | 0.000 |
|  | error | 0.000 | 8 | 0.000 |  |  |
|  | Total | 0.000 | 11 |  |  |  |
| Sperm motility (%) | Treatment | 1685.769 | 3 | 561.923 | 19.484 | 0.000 |
|  | error | 230.722 | 8 | 28.84 |  |  |
|  | Total | 1916.491 | 11 |  |  |  |
| Sperm COUNT (10^6^/ml) | Treatment | 2952.631 | 3 | 984.21 | 23.091 | 0.000 |
|  | error | 340.989 | 8 | 42.624 |  |  |
|  | Total | 3293.62 | 11 |  |  |  |
| Sperm viability (%) | Treatment | 1965.264 | 3 | 655.088 | 23.641 | 0.000 |
|  | error | 221.68 | 8 | 27.71 |  |  |
|  | Total | 2186.944 | 11 |  |  |  |
| Sperm abnormality (%) | Treatment | 291.129 | 3 | 97.043 | 44.877 | 0.000 |
|  | error | 17.299 | 8 | 2.162 |  |  |
|  | Total | 308.429 | 11 |  |  |  |
| FSH | Treatment | 21.014 | 3 | 7.005 | 40.349 | 0.000 |
|  | error | 1.389 | 8 | 0.174 |  |  |
|  | Total | 22.403 | 11 |  |  |  |
| LH | Treatment | 152.579 | 3 | 50.86 | 77.873 | 0.000 |
|  | error | 5.225 | 8 | 0.653 |  |  |
|  | Total | 157.804 | 11 |  |  |  |
| Testosterone | Treatment | 70.878 | 3 | 23.626 | 38.06 | 0.000 |
|  | error | 4.966 | 8 | 0.621 |  |  |
|  | Total | 75.844 | 11 |  |  |  |

Table 2. One-way ANOVA to study the effect of treatment type on the studied oxidative stress and COMET parameters.

|  |  | Sum of Squares | df | Mean Square | Fcalculated | P-value |
| --- | --- | --- | --- | --- | --- | --- |
| MDA | Treatment | 389.085 | 3 | 129.695 | 48.912 | 0.000 |
|  | error | 21.213 | 8 | 2.652 |  |  |
|  | Total | 410.298 | 11 |  |  |  |
| SOD | Treatment | 555502.2 | 3 | 185167.4 | 16.418 | 0.001 |
|  | error | 90228.32 | 8 | 11278.54 |  |  |
|  | Total | 645730.6 | 11 |  |  |  |
| CAT | Treatment | 41718.66 | 3 | 13906.22 | 34.678 | 0.000 |
|  | error | 3208.105 | 8 | 401.013 |  |  |
|  | Total | 44926.76 | 11 |  |  |  |
| GPx | Treatment | 63.181 | 3 | 21.06 | 20.044 | 0.000 |
|  | error | 8.406 | 8 | 1.051 |  |  |
|  | Total | 71.587 | 11 |  |  |  |
| TL | Treatment | 104.821 | 3 | 34.94 | 273.826 | 0.000 |
|  | error | 1.021 | 8 | 0.128 |  |  |
|  | Total | 105.841 | 11 |  |  |  |
| Tail DNA% | Treatment | 59.447 | 3 | 19.816 | 297.905 | 0.000 |
|  | error | 0.532 | 8 | 0.067 |  |  |
|  | Total | 59.979 | 11 |  |  |  |
| TM | Treatment | 6473.044 | 3 | 2157.681 | 370.337 | 0.000 |
|  | error | 46.61 | 8 | 5.826 |  |  |
|  | Total | 6519.655 | 11 |  |  |  |
